# A Review of Hybrid VLC/RF Networks: Features, Applications, and Future Directions

**DOI:** 10.3390/s23177545

**Published:** 2023-08-30

**Authors:** Lisandra Bravo Alvarez, Samuel Montejo-Sánchez, Lien Rodríguez-López, Cesar Azurdia-Meza, Gabriel Saavedra

**Affiliations:** 1 Department of Electrical Engineering, Universidad de Concepción, Edmundo Larenas 219, Concepción 4030000, Chile; lisanbravo@udec.cl (L.B.A.); gasaavedra@udec.cl (G.S.); 2 Programa Institucional de Fomento a la Investigación, Desarrollo e Innovación, Universidad Tecnológica Metropolitana, Ignacio Valdivieso 2409, Santiago 8940000, Chile; 3 Facultad de Ingeniería, Arquitectura y Diseño, Universidad San Sebastián, Lientur 1457, Concepción 4030000, Chile; lien.rodriguez@uss.cl; 4 Department of Electrical Engineering, Universidad de Chile, Santiago 8370451, Chile; cazurdia@ing.uchile.cl; 5 Millennium Institute for Research in Optics, Universidad de Concepción, Concepción 160-C, Chile

**Keywords:** hybrid networks, optical camera communication (OCC), radio frequency (RF), visible light communication (VLC), internet of things (IoT)

## Abstract

The expectation for communication systems beyond 5G/6G is to provide high reliability, high throughput, low latency, and high energy efficiency services. The integration between systems based on radio frequency (RF) and visible light communication (VLC) promises the design of hybrid systems capable of addressing and largely satisfying these requirements. Hybrid network design enables complementary cooperation without interference between the two technologies, thereby increasing the overall system data rate, improving load balancing, and reducing non-coverage areas. VLC/RF hybrid networks can offer reliable and efficient communication solutions for Internet of Things (IoT) applications such as smart lighting, location-based services, home automation, smart healthcare, and industrial IoT. Therefore, hybrid VLC/RF networks are key technologies for next-generation communication systems. In this paper, a comprehensive state-of-the-art study of hybrid VLC/RF networks is carried out, divided into four areas. First, indoor scenarios are studied considering lighting requirements, hybrid channel models, load balancing, resource allocation, and hybrid network topologies. Second, the characteristics and implementation of these hybrid networks in outdoor scenarios with adverse conditions are analyzed. Third, we address the main applications of hybrid VLC/RF networks in technological, economic, and socio-environmental domains. Finally, we outline the main challenges and future research lines of hybrid VLC/RF networks.

## 1. Introduction

In recent years, the limitations of the radio frequency (RF) spectrum for mobile communications have become very evident [1]. This is a great challenge to overcome in light of the traffic load demands associated with fifth-generation (5G) mobile communication, with alarming projections beyond 5G and into the sixth generation (6G). Due to these limitations, the dense deployment of RF access points leads to high competition for available channels [2], which entails degradation of the quality of service (QoS).

As an alternative solution, visible light communication (VLC) has been proposed, an optical wireless communication technology that uses the visible light spectrum with wavelengths between 375 and 780 nm [3]. This technology uses light-emitting diodes (LEDs) that produce incoherent light to illuminate and transmit data simultaneously, achieving high data rates of hundreds of megabits per second [4]. With the goal of secure and efficient communications, the VLC Consortium (VLCC) was established to promote and standardize VLC technology in 2007 [5]. VLC is characterized by high security, as information cannot be filtered [6]. In addition, it is immune to RF interference, meaning that the system can be used freely in environments sensitive to electromagnetic signals [7]. VLC has experienced rapid development and has attracted much interest from researchers [8]. In 2011, the Visible Light Communications Task Group created the IEEE 802.15.7 standard [9] that establishes three physical (PHY) VLC operation modes, as shown in Table 1. The three PHY modes can coexist with each other, thereby mitigating LED flicker and reducing dimming [10]. In 2019, the ITU-T Telecommunication Standardization Sector finalized recommendation G.9991 that established the first commercially ready light fidelity (Li-Fi) standard [11]. Both IEEE 802.15.7 and G.9991 have now converged into IEEE 802.11bb to refine the medium access control (MAC) and physical layer (PHY) of VLC [12]. One of the essential features of the IEEE 802.11bb standard [13] is that there is a single medium access control (MAC) sublayer common to all physical layers. This feature enables easier interoperability between the different physical layers, making cooperation between RF and visible light communications technologies possible [2].

On the other hand, the implementation of the B5G cellular networks has brought with it an increase in mobile traffic with applications such as virtual reality, augmented reality, and online video games [14]. Ultra-high capacity wireless connectivity is considered a key technology to support end-to-end delivery in the era of beyond 5G and 6G [15,16]. Therefore, there is a need for a network infrastructure capable of supporting multiple new services that demand ultra-high transmission speeds, device connectivity, and high quality of experience (QoE) [17].

In the last few years, researchers have shown great interest in hybrid VLC/RF systems, and this type of network is expected to be the basis for the development of B5G and the next generations of cellular networks [18,19,20]. Hybrid VLC/RF networks are presented as a complementary technology capable of realizing cooperation between RF and VLC networks without interfering with each other [18]. They are able to significantly increase the system data rate and achieve better load balancing by distributing the data load between the two networks [19]. Additionally, they have a very low implementation cost due to the extensive use of LEDs in lighting infrastructure around the globe that can be reused for mobile communication [20].

**Figure 1:**
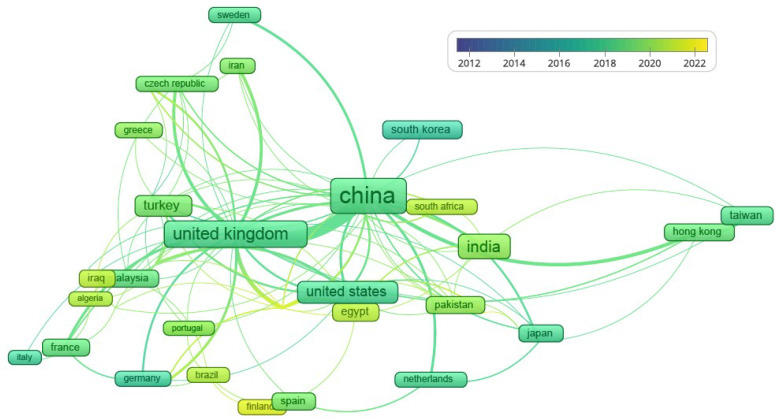
Bibliometric analysis of the VLC/RF networks studies by countries.

In this sense, a complete search was carried out in the Scopus database [21] using the keywords of Visible Light Communication/Ratio Frequency. A total of 727 English-language papers were collected from the years 2012–2022. A bibliometric analysis was performed using the VOSviewer software tool [22] (see Figure 1). The analyzed items were the published documents, keywords, country distribution, and affiliation.

Figure 1 shows a clear increase in the number of research papers published in the last decade in the field of hybrid VLC/RF networks, which demonstrates the growing scientific interest in this area of research. The countries appearing in the search according to the affiliation of the main authors were China (257), the United Kingdom (99), India (92), the USA (54), and Turkey (44). The universities with more than five publications related to the VLC/RF field were Ozyegin University, Istanbul; Southeast University, China; and Northumbria University, United Kingdom. This analysis shows that research related to this area is greater in the northern hemisphere than in the southern hemisphere, Chile being one of the countries with the lowest number of publications in this area (2).

The emergent Internet of Things (IoT) has boosted the need for innovative communication solutions that provide reliable and efficient data transmission methods. An intelligent, efficient, and reliable system can be obtained by combining VLC and RF technologies [23]. This integrated system can support various IoT applications, such as smart lighting, home automation, e-healthcare, and industrial IoT. For example, in smart lighting the VLC network can be used to provide high-speed communication through luminaries while the RF network can be used to control luminaries from a remote location [24]. Similarly, in e-healthcare applications the VLC network can provide reliable communications between medical devices, while the RF network can transmit data to healthcare providers or emergency services. Therefore, the implementation of hybrid VLC/RF networks offers a reliable and efficient solution for IoT applications, especially indoors [25].

Due to the great interest shown in hybrid VLC/RF networks, several authors have conducted reviews of the history of these networks [2,17,26,27,28,29]. However, the dynamic and changing characteristics depending on the deployment scenario and the extensive evolution of VLC/RF networks in recent times have produced the need to delve deeper into issues that have not been fully analyzed by the existing reviews. Table 2 compares this work with the most prominent reviews in the last ten years relating to hybrid VLC/RF networks. We used the markers ⋆ and ★ to represent partially and deeply analyzed topics, respectively. In our criterion, we mark as partially analyzed those topics that were only commented on in a general way without going deeper into characteristics, problems, and possible solutions. On the other hand, the papers marked as deeply analyzed are those that we consider to address each of the topics mentioned in detail, providing characteristics of the technologies, pending challenges, an analysis of what has been achieved by other authors, and possible solutions that can be carried out to optimize VLC/RF networks.

Therefore, in this work an extensive study of the state of the art of hybrid VLC/RF networks in ideal indoor scenarios in the scientific literature is carried out and the characteristics and implementation of these networks in outdoor scenarios with harsh conditions are analyzed. The main applications of hybrid VLC/RF networks in the technological, economic, and social-environmental fields are considered. In addition, the main challenges faced by hybrid VLC/RF networks and future research directions are discussed.

The rest of this paper is organized as follows. In Section 2, the state of the art of hybrid VLC/RF networks in indoor scenarios with ideal conditions is reviewed, including analysis of aspects such as lighting requirements, hybrid channel model, load balancing, network selection, and resource allocation, and hybrid network topologies. In Section 3, a review of the state of the art of hybrid VLC/RF networks in outdoor scenarios with harsh conditions is presented. Section 4 analyzes the possible applications of hybrid VLC/RF networks in the technological, economic, and social-environmental fields. Section 5 establishes the main challenges and future directions of these hybrid networks. Finally, the conclusions of this review are presented in Section 6.

## 2. Hybrid VLC/RF Networks in Indoor Scenarios under Ideal Conditions

**Figure 2:**
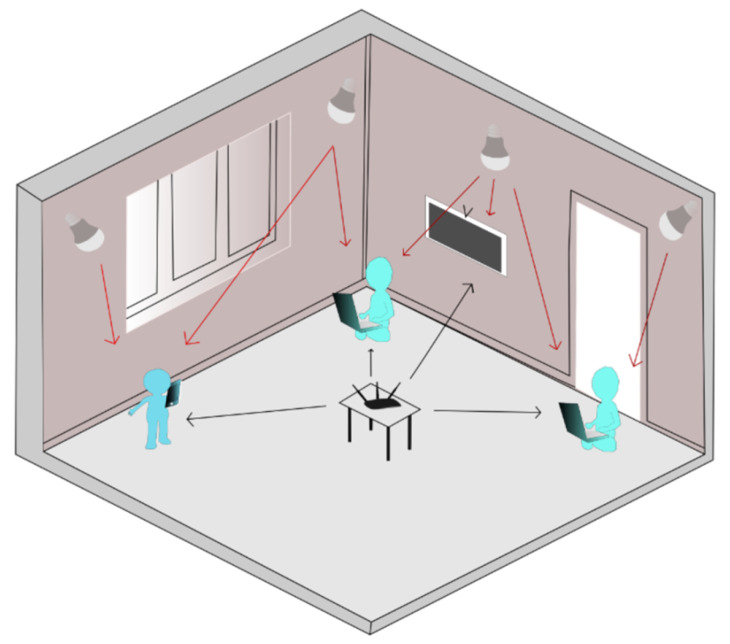
Hybrid VLC/RF network in an indoor scenario.

In this section, a review of the state-of-the-art of hybrid VLC/RF networks in indoor scenarios with ideal conditions is conducted. A hybrid VLC/RF network in an indoor scenario can be composed of LED arrays located on the ceiling or walls of a room that provides the VLC connection and one or several radio frequency access points (APs) that provide the RF connection. Figure 2 shows an example of a hybrid VLC/RF network in an indoor scenario. Here, users inside the room can simultaneously connect to both networks using the VLC connection through the existing lighting and wireless access to the AP for the RF connection. Being an enclosed room, the illumination is provided by LED lights only, and there is no interference from outside lighting or ambient noise to affect the connection.

### 2.1. Lighting Requirements

**Figure 3:**
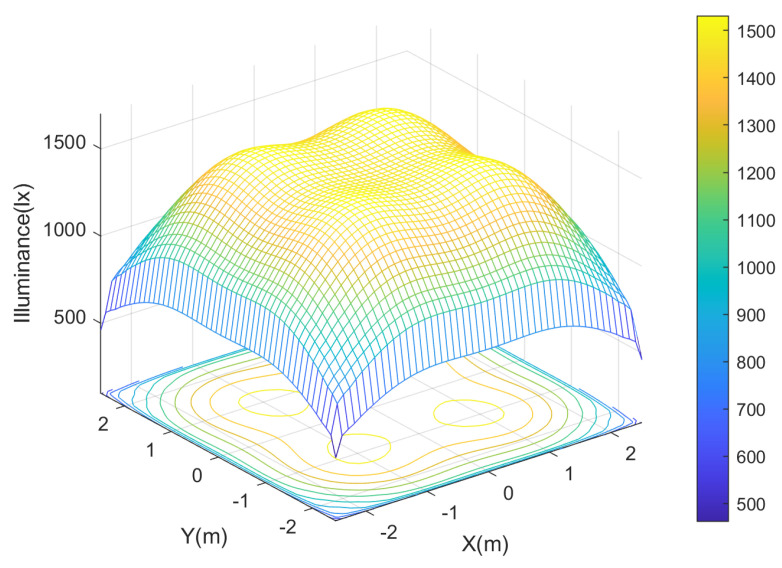
Illuminance level of a $5\;m^{2}$ room with 4 LED lights.

Through proper room lighting design, the energy efficiency of the VLC/RF hybrid network can be improved. The goal is to achieve the desired lighting pattern with minimum energy consumption. This requires optimizing the placement and control of LED output power levels. Lighting quality is essential in these hybrid networks because all information and electronic signals are transmitted through light [32]. A bad lighting design can affect the quality of the signal; therefore, it is necessary to take into account different aspects such as the distance between the devices, the intensity of illumination, environmental interference, and power management. The illumination level stipulated by the International Organization for Standardization (ISO) for an indoor environments is 300 to 1500 lx. Generally, the greatest illumination is in the center of the room rather than in the corners, so careful design is required to provide uniform illumination throughout the room. In [2], a lighting scheme with the following requirements is established: $ilum\left(x,y\right)=\sum_{i=1}^{n_{LEDs}}\frac{I_{0}}{d_{i}^{2}}{cos}^{l}\left(\theta_{i}\right)cos\left(\phi_{i}\right)$, where $I_{0}$ is the illumination intensity of the LEDs, and $d_{i}$ is the Euclidean distance between the ith LED and the arbitrary location (x,y) of the user. The number of LEDs is denoted by $n_{LEDs}$, and *l* is the Lambertian emission order. The radiation and angle incidence at the (x,y) location of the user is denoted by $\theta_{i}$ y $\phi_{i}$, respectively. Figure 3 shows an example of lighting inside a $5\;m^{2}$ room illuminated by 4 LEDs placed uniformly on the ceiling. As the intensity of light increases, so does the data rate. However, the relationship between illumination and throughput is not always linear, as interference between different lighting devices and physical obstacles can affect link quality and reduce throughput. To reduce interference between devices, it is necessary that lighting and data transmission share the same frequency spectrum in the VLC network.

The versatility of the lighting system lies in its ability to serve as an ideal vehicle for VLC technology, paving the way for the development of various Internet of Things (IoT) applications. By blending the potential of smart sensors with lighting, it becomes possible to create cutting-edge IoT solutions that are both unique and resourceful [33]. Notably, the lighting system can revolutionize several urban applications, from creating smart homes to building energy-efficient offices and even revolutionizing urban infrastructure through smart streetlights. This combination of light and technology opens up endless possibilities in the IoT realm, enabling the development of novel innovative solutions that cater to myriad industrial and societal needs [34].

In [35], an algorithm based on a fast game theory for optimizing energy efficiency under illumination constraints was presented. The authors formulated a generalized Nash equilibrium problem in which each user maximizes its own VLC energy efficiency while subject to the shared illumination constraint. In [36], an electrical and optical power allocation scheme was proposed taking into account the lighting constraints in order to maximize the multi-user sum rate (throughput). It was shown that with increasing luminous flux the maximum summation rate exhibits a downward parabolic trend due to the limited dynamic range of LEDs. Alternatively, a higher data rate can be achieved with a higher correlated color temperature. In [37], a simple approach to the illumination design problem based on the virtual LED concept was presented. The authors formulated an optimization problem with constraints to minimize energy consumption while maintaining near-uniform illumination throughout the room. In addition, they derived the number of LEDs and the luminous intensities of the LEDs required to achieve the desired lighting constraint.

### 2.2. Hybrid Channel Model

In an indoor scenario, the impulse response of a VLC channel is modeled through a Geometric Based Model, which can be approximated for simplicity to a Lambertian model, which depends on half the beamwidth angle of the transmitting LED [38]. The total channel impulse response is provided by the sum of the line-of-sight (LOS) impulse response and the non-line-of-sight (NLOS) impulse response [39] using recursive methods. When the LEDs are very close to the receiver, a LOS link is achieved, promoting a channel model without temporal dispersion. When there are multiple signal reflections due to walls or obstacles within the room or the distance between the emitter and receiver is very large, NLOS links are created. To calculate the impulse response of these links, it is necessary to take into account the signal tails caused by time dispersion and multiple paths. This is because the information transmitted by the LED may reflect off stage walls or other blocking object before reaching the receiver [40].

Because LEDs produce light with non-coherent characteristics and have a limited dynamic range, the most conducive modulation scheme for VLC is intensity modulation (IM)/direct detection (DD) [41]. When an optical OFDM signal is used in an IM/DD system, intensity modulation of the carrier signal is performed. Therefore, the transmitted signal must be real and of positive value in the time domain. Consequently, it is necessary to use real transformations such as the discrete cosine transform (DCT) or the fast Walsh Hadamard transform (FWHT), among others, to obtain a real signal. If the optical OFDM signal cannot be matched to the linear region of the LED voltage–current relationship, clipping of the signal occurs, causing nonlinear distortions. To overcome the distortions induced by this nonlinearity, peak-to-average power ratio (PAPR) reduction techniques or predistortion techniques must be used [42].

An electromagnetic wave incident on an object can be reflected, refracted, scattered, diffracted, or absorbed by it. This depends on the frequency of the incident wave and the physicochemical properties of the object. This interaction determines the level of opacity or transparency of the object at different wavelengths. When white light falls on a surface, certain wavelengths are absorbed while others are reflected, determining the object’s color. Opaque objects such as wood, metal, and plastic restrict the propagation of visible light [43]; therefore, when designing a VLC communication channel the behavior of visible light wavelengths interacting with such materials strongly influences the decision-making process. Conversely, an object can be transparent at certain wavelengths, allowing light to pass through it easily.

On the other hand, the RF communication channel model consists of the path loss model and the spatial channel model, usually composed of the lumped channel impulse responses and angular dispersion statistics [44]. In an indoor scenario, the received signal arrives through multiple simultaneous paths that are vectorially combined to obtain a resulting oscillatory signal. This multi-path can be caused by phenomena such as reflection, refraction, diffraction, or scattering. The most common way to include these phenomena is by means of the channel impulse response model, which is presented in a temporal way, as it can have discrete values or be a stochastic variable [45]. Due to the symmetry properties of the RF channel, the RF channel model can be Hermitian. The ratio between the imaginary part and the real part of the channel impulse response defines the statistical distribution in which the signal fades follow at each coefficient, that is, each delay can be considered a different variable, typically Rayleigh, Rice, or simply Gaussian depending on whether or not there is a predominant component [46]. The possibility of Doppler effects due to mobility between transmitter and receiver must be taken into account. In addition, if an orthogonal frequency division multiplexing with a multiple inputs–multiple outputs (OFDM-MIMO) system is used, spatial diversity must be taken into account, making it necessary to introduce parameters at one end related to the correlation between the different antennas [47].

**Figure 4:**
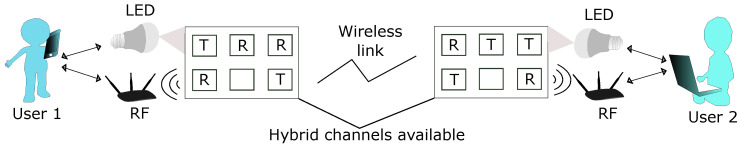
Hybrid model of a VLC/RF communication channel.

Table 3 shows a comparison of the communication channel characteristics of VLC and RF networks. Although these two technologies have very different communication channel models, it is possible to design a hybrid model because there is no interference between the two networks. In [48], a robust channel model of a hybrid VLC/RF system was developed for optimal channel selection. Figure 4 shows a hybrid model of a VLC/RF communication channel composed of mobile terminals at each end equipped with hybrid VLC/RF frequency bands. The RF channel has a Hermitian model with WLAN frequencies (2.4 GHz/5 GHz), while the VLC channel approximates a Lambertian model with frequency bands on the order of THz. The wireless communication channels are shared simultaneously between the two technologies to transmit (T) and receive (R) information. The authors of [49] described a hybrid model of an imperfect VLC/RF channel, which they used to analyze the energy efficiency of these hybrid systems. Among the imperfections of the hybrid channel, they took into account the uncertainty caused by noise and quantization errors in the digital–analog process.

Due to the presence of hybrid frequency bands and the dynamic conditions of the hybrid communication channel, optimal channel design is a very important and difficult challenge for VLC/RF systems. Optimizing the transmission channel of the VLC/RF network is essential for the development of IoT systems because smart devices often require a stable connection for proper operation. A reliable and robust connection can help extend the lifetime of IoT devices and their applicability.

### 2.3. Load Balancing

One of the most important elements in hybrid networks is load balancing, as it manages traffic and resources in the network. Load balancing helps to evenly distribute a load of operations and traffic among each device connected to the hybrid network. This reduces network congestion, prevents overheating of devices, and improves performance in terms of speed, latency, and reliability [50].

Part of the literature on VLC/RF systems focuses on the development of algorithms to optimize network load balancing. The goal is for each user to find the best VLC or RF access point which provides the highest data rate depending on the location and mobility of each user. In [51], a protocol combined with horizontal and vertical handover mechanisms was proposed for a mobile terminal to solve the mobility of users between access points and the OFDMA system. In addition, a VLC network scheme was presented to solve multi-user access problems at each access point in the network. The authors of [52] proposed a load-balancing algorithm to manage hybrid VLC/RF network resources and determine the association of users to each system, and additionally proposed a decentralized optimization method based on Lagrangian multipliers. In [53], a bidirectional selection handover algorithm that takes load balancing into account was developed to improve the overall performance of the hybrid system. Such an algorithm selects the suitable target AP based on the hybrid technique of fuzzy analytic hierarchy process and order preference technique based on the similarity to the ideal solution.

**Figure 5:**
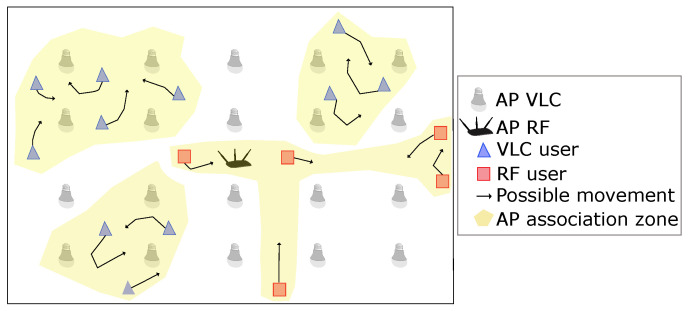
Load balancing scheme of a hybrid VLC/RF network in an indoor scenario.

Figure 5 shows a load balancing scheme of a hybrid VLC/RF network in an indoor scenario. Here, users are associated with an access point, either VLC (red triangles) or RF (blue squares), depending on the location and possible movement of each one. The association of users with the AP only takes place within the association zone (yellow shadow). In order to save network resources, access points outside of this zone (far from the users) do not transmit.

Another part of the research focuses on the objective of minimizing the handover latency and increasing the overall network throughput using load-balancing techniques such as Handover. Handover in hybrid VLC/RF networks is the process through which a user switches from one network to another without losing connectivity. Thus, if a user connected to the VLC network moves to an area without access to the VLC network, the device automatically connects to the available RF network [54]. Similarly, if the user returns to a VLC access zone, the device automatically reconnects to the VLC network without losing connectivity. During this process, the quality of the received signal and the availability of the networks must be monitored to ensure that the connection transfer from one network to another is optimal. In [55], the authors proposed a dynamic handover scheme based on fuzzy logic with the objective of reducing the handover overhead. This scheme takes into account channel state information, user rate, and desired data rate to determine whether or not to request a handover. If a mobile terminal moves from one access point to another, a handover of the mobile load occurs between the access points. This handover can be classified as horizontal if the change occurs intra-system or vertical if the change occurs inter-system. Vertical handover is a fundamental type of handover to improve the performance of hybrid VLC/RF networks. The authors of [56] formulated a vertical handover scheme as a Markov decision process problem. Moreover, they adopted a dynamic approach to obtain a compromise between the switching cost and the delay requirement. On the other hand, the authors of [57] proposed an algorithm based on decision selection, taking into account dynamic network parameters and actual traffic preferences. The degree of rationality of each decision was calculated by combining the results of the analytical hierarchy process and a two-person cooperative game model. Recently, the authors of [58] proposed an automated vertical handover algorithm for VLC-WiFi networks using the received signal strength values. This algorithm selects the next most likely base station to transmit based on the hidden Markov model.

Horizontal handover is a fundamental technique in hybrid VLC/RF networks. If a user is moving from one location to another connected to one of the two networks of the system (VLC or RF), it is necessary to perform a horizontal handover to move the mobile load to the next access point of the same technology. In [59], an adaptive horizontal and vertical handover mechanism for hybrid VLC/MMW systems was proposed. The mechanism was based on a centralized system architecture and used the signal-to-interference plus noise ratio levels of the APs as a performance metric. The authors of [60] proposed a solution to the horizontal and vertical handover of a VLC/WiFi network to address problems due to user mobility, obstacles, and shadowing.

Table 4 shows a summary of studies carried out in the literature on load balancing in hybrid VLC/RF networks in the last ten years. Although this topic has been extensively studied in recent years, the constant evolution and dynamics of cellular networks make it necessary to constantly update load-balancing algorithms.

### 2.4. Network Selection and Resource Allocation

The main objective of communication networks is to provide users with personalized demand, high quality of service (QoS), and high overall system performance. Achieving this goal is a constant challenge in hybrid VLC/RF networks because of the need for an elaborate balance between user demand and network resources [61]. Another fundamental challenge in these networks is the problem of associating mobile terminals to each base station for packet transfer due to the density of access points, their heterogeneity, differences in coverage area, and the limitation of available resources [62].

For all these reasons, it is very important to realize an approach to address the problem of network association and resource allocation and to obtain better information about the users and resources in the environment. The authors of [63] proposed a network selection scheme for a hybrid VLC/RF system in an indoor scenario, taking into account the context-aware information of asymmetric downlink and uplink characteristics and network performance. In addition, a context-dependent learning algorithm was proposed based on the information about the location of the users and the type of traffic they require. In [64], the authors proposed a knowledge transfer reinforcement learning algorithm to perform network selection in a hybrid VLC/RF system. This algorithm reveals and exploits contextual information about networks, load distribution, and network traffic characteristics. The authors of [65] showed another approach to network selection by designing a fuzzy logic model with relatively low computational complexity, in which all users to be connected to the RF network are first selected and then all remaining users are assigned to the VLC network. In [66], a network selection process was proposed for a hybrid system composed of VLC and RF, in which the transmitter sends the transmission data over the link that best guarantees the quality of service. The average arrival data rate at the transmitter buffer and the non-asynchronous limits of the buffer delay are used to evaluate the performance of the proposed algorithm.

**Table 4:** Summary of the studies of load balancing in hybrid VLC/RF networks.

Ref	Year	Hybrid Network	Contributions
[51]	2014	VLC/OFDM	A combined protocol with horizontal and vertical handover mechanisms is proposed for the mobile terminal to solve the mobility of users between different access points and the OFDMA system. A VLC network scheme and its frame format are presented to solve multi-user access problems at each access point. A new metric is defined to evaluate the capacity of the hybrid network based on the assumption of homogeneous Poisson point process distribution of users.
[67]	2015	LIFI/WIFI	Load balancing is studied taking into account user mobility and handover signaling overhead. A dynamic load balancing scheme is proposed considering system throughput and fairness. The service areas of different APs are studied and the performance of each AP is analyzed using the proposed load-balancing scheme.
[56]	2015	VLC/WIFI	An efficient vertical handover scheme is investigated by formulating it as a Markov decision process problem. A dynamic approach is adapted to obtain a compromise between the switching cost and the delay requirement. The proposed scheme determines whether to perform vertical handover given the queue length and the wireless optical channel condition.
[68]	2015	RF/VLC	A cooperative load balancing that achieves proportional fairness using centralized and distributed resource allocation algorithms is studied. Performance is analyzed in terms of both throughput and fairness under various cell formation scenarios.
[55]	2016	LIFI/RF	A dynamic handover scheme based on fuzzy logic is proposed, which uses channel state information, user rate and desired data rate to determine if the handover is necessary. The objective is to reduce the handover overhead by properly assigning users to the RF or LiFi access point.
[57]	2017	VLC/Femto	An approach that takes into account multiple attributes, including dynamic network parameters and actual traffic preferences, is performed using the analytical hierarchy process. The degree of rationality of each decision is calculated by combining the results and the decision with the highest degree of rationality is selected.
[69]	2017	LIFI/RF	A dynamic load balancing scheme that takes into account the handover overhead is proposed to improve the overall system performance. A joint optimization algorithm and a separate optimization algorithm, which jointly and separately optimize the access point allocation and resource allocation, are proposed.
[50]	2018	VLC/RF	An iterative algorithm is proposed to distribute users into APs and to distribute the powers of the APs to their users. An optimization problem is formulated to allocate the power of each AP to the connected users in order to maximize the total achievable data rate. Two approaches are proposed to perform joint AP and load balancing: a main approach that considers the exact interference information for all users, and a suboptimal approach that aims to decrease the complexity of the first approach by considering only the approximate interference information of the users.
[70]	2018	LIFI/RF	A handover algorithm based on the received signal strength indicator is considered. A theoretical analysis of the handover probability based on a Markov chain model is presented.
[71]	2019	LIFI/WIFI	An overview of the concept of hybrid handover in LiFi/WFi networks is given and related work done in this field is discussed.
[52]	2020	VLC/RF	A load balancing algorithm is proposed to manage the hybrid network resources and determine the association of users to each system. A decentralized optimization method based on Lagrangian multipliers is proposed.
[53]	2021	VLC/RF	A bidirectional selection handover algorithm is developed that takes load balancing into account to improve system performance. The appropriate destination AP is selected based on the hybrid technique of fuzzy analytic hierarchy process and order preference technique by similarity to the ideal solution for each user that needs to handover.
[72]	2021	VLC/WLAN	A vertical handover technique that uses the user’s location information to make a handover decision is proposed. The proposed algorithm is contrasted with the immediate vertical handover algorithm and the permanent vertical handover algorithm with two different dwell times.
[26]	2022	VLC/MMW	An event-triggered adaptive handover mechanism for centralized hybrid systems is proposed. The handover variables are self-adjusted using rule-based algorithms with the number of connected users and the data transmission rate as input variables.
[60]	2022	LIFI/WIFI	A higher layer handover solution known as “Li-Wi” is proposed to address handover issues due to mobility, shadows and obstacles. Robust connectivity with shorter handover outage duration and high network throughput is provided.

In hybrid VLC/RF networks, resource allocation is closely related to network selection, as the selection of one of the two networks depends on the allocation of resources to each network, just as the allocation of resources to each network has a significant impact on network selection to optimize system performance [73]. An optimal combination of resource allocation and network selection enables overall system performance in terms of data rate, delay, and energy efficiency. Several types of research on resource allocation in hybrid VLC/RF networks are found in the literature [20,74,75,76]. A study of resource allocation in two different scenarios of hybrid VLC/RF networks to maximize user throughput was recently performed in [74]. In the first scenario, a hybrid system was modeled in which VLC is powered by light waves to radiate only optical energy and the throughput maximization problem is formulated by optimizing the uplink time allocations. In the second scenario, time-switching is performed to simultaneously transfer information and power over the lightwave of the VLC network and a multi-objective optimization problem is formulated through a set of Pareto-optimal resource allocation policies. On the other hand, in [20] a resource allocation scheme that maximizes the data rate, taking into account user fairness was proposed for a VLC/RF network, with both networks served by a common backhaul and the channel state information able to be either perfect or imperfect. The authors of [75] developed a resource allocation algorithm with the objective of maximizing the energy efficiency (throughput per unit power) of the hybrid system. The results obtained by simulating the algorithm show that the energy efficiency can be increased if all the VLC access points are evenly distributed within the scenario. Lastly, in [76] a centralized algorithm for joint downlink resource allocation in a hybrid VLC/RF network was developed.

### 2.5. Hybrid Network Topologies

**Figure 6:**
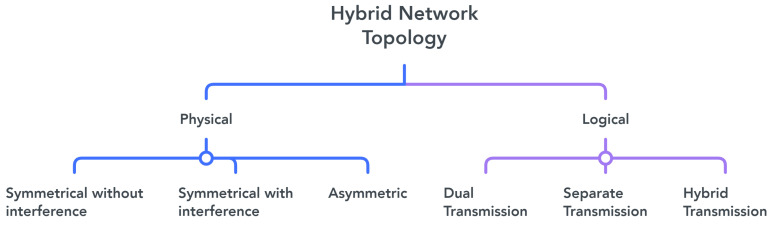
Types of hybrid topologies in the literature.

Network topologies are primarily used to describe the physical or logical structure of a network, i.e., the topology defines how and where the various devices in the network are connected. The correct design of the topology of a hybrid network is very important because it allows for optimization of the location and interconnection between the different access points of the network [77]. The main topologies designed for hybrid VLC/RF networks are separated into six major groups: symmetric with interference, symmetric without interference, asymmetric, dual transmission, separate transmission, and hybrid transmission, as shown in Figure 6.

**Figure 7:**
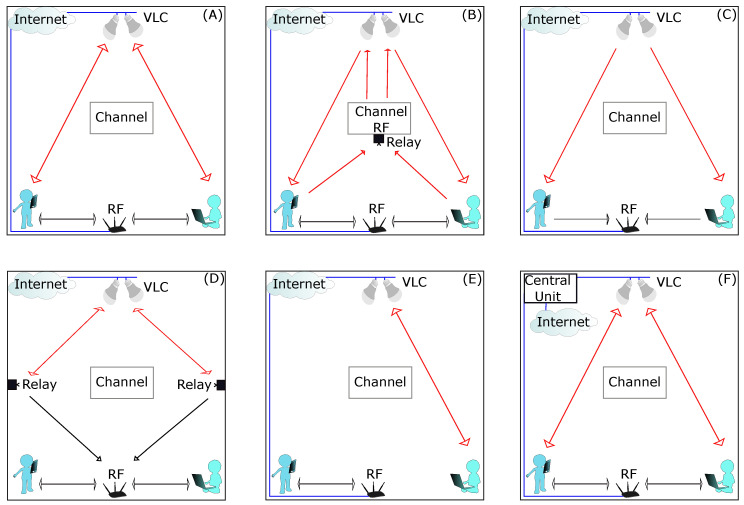
Different hybrid network topologies: (**A**) symmetrical without interference, (**B**) symmetric with interference, (**C**) asymmetric, (**D**) dual transmission, (**E**) separate transmission, (**F**) hybrid transmission.

Symmetrical without interference is a topology in which both networks are independent. Both the VLC network and the RF network can be used for downlink and uplink connection, as shown in Figure 7A. There is no interference between the networks [56,63,65,67,69,75,78,79,80,81,82,83,84,85,86].

In symmetric with interference topologies, the VLC and RF networks are linked in the uplink; therefore, they are not independent and interference occurs between them. Figure 7B shows a schematic of a symmetrical topology with interference. In the downlink, both networks can be used simultaneously without interference, while in the uplink only the RF network is used to transmit information from both networks, which causes interference between RF users and VLC users transmitting over the RF network [87,88,89,90,91].

Figure 7C shows an asymmetric topology scheme. The downlink and uplink connections are routed in different directions for the two networks. The VLC network is only used for the downlink connection, while the RF network is only used for the uplink [52,77,92,93,94,95,96,97,98,99,100,101,102,103,104].

In dual transmission topologies only one of the two networks can be connected. The VLC network is used for the connection between the VLC access points and the RF access point via a relay, while the RF network is used for the connection between the RF access point and the users. Both networks transmit on the downlink and uplink, as shown in Figure 7D [18,105,106,107,108,109,110].

In separate transmission, the two networks are separated into independent connections. As shown in Figure 7E, users can choose between one of the two networks depending on the location and movement of each user as well as the SNR of the links. While this topology has great flexibility for users, it is difficult to control and is not optimal [49,111,112,113,114,115,116,117,118,119,120,121,122,123].

Figure 7F shows a hybrid transmission topology, in which both networks are interconnected by a control unit. Depending on the location of the users and the network conditions, the control unit allocates network resources to each user, ensuring fairness. The connection can be made by both networks in both the downstream and upstream directions [50,68,78,124,125,126,127,128,129,130,131,132,133,134,135].

## 3. Hybrid VLC/RF Networks in Outdoor Scenarios with Harsh Conditions

In an outdoor scenario with harsh conditions (e.g., sunlight, rain, dust, wind) the communication channel of hybrid VLC/RF networks becomes very complex and dynamic. These conditions distort the VLC optical beam, causing amplitude and phase fluctuations along with optical losses. The level of loss of the VLC signal is higher than the losses suffered by the RF network, as the VLC signal is very sensitive to atmospheric conditions such as sun, rain, and fog [136]. There are several factors (scattering, absorption, non-alignment, and turbulence) that can cause signal loss in both the VLC and RF networks, which complicate the design of the hybrid communication channel model [137]. Among the most important phenomena responsible for signal loss in VLC/RF networks are:

- Scattering corresponds to an angular spread of the optical field, which is independent of frequency and wavelength in certain cases. It can be caused by solar radiation, rain, or particles and objects in the environment, which create a divergence in the transmission beam and cause the size of the received transmission beam to be larger than the receiver aperture, resulting in part of the signals being lost.
- Absorption takes place when a collision occurs between the photons of the propagating beam of the VLC network and the particles or molecules existing in the environment. The absorption coefficient depends on the type of molecules in the air and their concentration. There are areas of minimum absorption in the atmosphere called transmission windows. One way to avoid or decrease this phenomenon is to select the wavelengths of the grid to be within the wavelengths of the transmission window.
- The distance between the transmitter and receiver in an outdoor scenario can cause non-alignment of the transmitter and receiver, resulting in a loss of signal power. Non-alignment between the transmitter and receiver can be due to the presence of obstacles, buildings, and trees, as well as changes in the weather. As the distance between the transmitter and receiver increases, these factors must be taken into account.
- Ambient turbulence corresponds to a divergence in the optical signal, which can be caused by random temperature variations, solar radiation, increased wind speed, and atmospheric pressure. Divergence caused by turbulence during transmission results in random variations of signal amplitude and phase, causing fading of the received power and decreased performance of hybrid systems.
- In an outdoor scenario, multiple sources of noise and interference can affect hybrid VLC/RF networks, including shot noise, thermal noise, and environmental interference. Among these, sunlight is considered the most serious co-channel interference source for the VLC network, and thus for the hybrid network, as it can significantly reduce the SINR of the system. One way to reduce the level of sunlight interference in the VLC network is to use lenses and filters on the receivers; however, this is not an optimal solution.

The harsh conditions in outdoor scenarios are important factors to take into account in the design of hybrid VLC/RF networks. A study of the degrading effects of physical phenomena on the quality of VLC communications in an industrial environment, where dust, other particles, and artificial light sources increase signal attenuation and cause optical interference, was carried out in [138]. In such cases, it is necessary to design and implement a robust model capable of considering the existing environmental conditions in order to improve the overall performance of the hybrid system [139]. The use of transmitters and receivers with complementary metal oxide semiconductor-based cameras and image sensors is a robust way to realize VLC network connection in outdoor scenarios, as complementary metal oxide semiconductors are capable of converting the modulated optical signal into an electrical waveform. As in indoor scenarios, a hybrid VLC/RF network can be implemented through different topologies in outdoor scenarios, mainly depending on the type of VLC network link, i.e., line-of-sight (LOS) or non-line-of-sight (NLOS). A very suitable scheme for an outdoor scenario with a line-of-sight link is the Point-to-Point topology. In this topology, the transmitters and receivers are in a fixed position and oriented towards each other. Otherwise, if the link is NLOS, a fuzzy topology can be used in which the transmitter and receiver are not require to be aligned. In this case, a receiver with a panoramic Lambertian pattern that can rotate within the radiation field of the transmitter is used to receive the signal [140].

**Figure 8:**
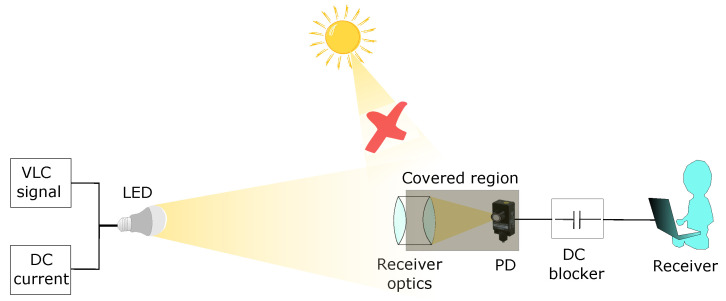
General schematic of a VLC transceiver system with an optical filter.

One of the main challenges in hybrid VLC/RF networks stems from difficult outdoor conditions, mainly due to the high mobility of background noise caused by sunlight or other artificial light sources. A study of the impact of sunlight noise sources on the packet delivery rate in a VLC network was carried out in [141]. The results showed that when the sun is not in the receiver’s field of view (FOV) a packet delivery rate of 100% can be achieved for distances of less than 100 meters. On the other hand, Turan performed a time-domain characterization of the VLC channel by conducting real experiments in a static outdoor scenario during different times of the day [142]. The results showed that sunlight both affects the effective useful bandwidth of the VLC channel and reduces the channel gain when direct sunlight is present in the receiver FOV. To counteract the adverse effects of sunlight on the VLC channel, the receiver FOV can be optimized by adjusting the receiving angle. It should be noted that FOV adjustment is usually a compromise between noise reduction and the possible reception angle. To avoid reducing the receiver FOV too much, in [143] the use of a high-pass filter was proposed to filter the sunlight intensity in a one-channel VLC experiment. This filter was composed of a biconvex lens adaptive optical receiver, allowing high reduction of background noise without affecting the FOV angle. In addition, the authors coated the entire area from the optical receiver to the photodiode in order to block unwanted light angles, as shown in Figure 8. Finally, they added a DC blocker after the photodiode to block strong DC components. Experiments showed that data transmission is possible up to a distance of 40 m under perfect lighting conditions; however, the connection distance decreases in strong sunlight.

In visible light communication, due to the propagation and directionality characteristics of light it is important to establish a clear line-of-sight (LOS) link between the transmitter and receiver. However, maintaining this LOS link becomes a challenge when users are in motion. This is especially true in outdoor environments, where light beams can travel long distances and reflected components lose energy, making reliable communication difficult. In [144], the authors suggested that non-line-of-sight (NLOS) communication via ground reflections can be advantageous in VLC. The effectiveness of NLOS communication depends on factors such as weather conditions and ground surface materials, which influence ground reflectivity. Unlike traditional wireless communication, VLC communication does not suffer from multipath fading caused by NLOS reflections. This is due to the inherent spatial diversity resulting from the carrier wavelength of visible light waves, which is significantly smaller compared to the size of typical receivers [43].

**Figure 9:**
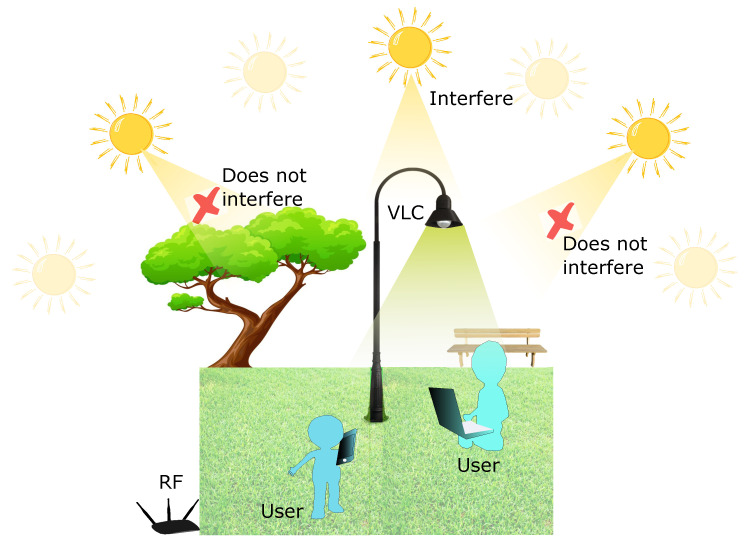
VLC/RF hybrid network in an outdoor scenario for different sun positions.

Based on the conditions of the outdoor VLC channel and the solutions proposed by [141,142,143], we propose designing a hybrid outdoor VLC/RF network as shown in Figure 9. This hybrid VLC/RF network can provide service to users through both networks at times when the solar intensity direction does not coincide with the direct FOV angle, thereby taking advantage of the multiple benefits of hybrid networks. At times when it is not possible to connect through the VLC network due to saturation of the photodiode by high background noise caused by sunlight, the service can be provided only by the RF network to ensure that users are not left without connection at any time. At night, the VLC network can be affected by artificial light sources such as lights used by cars and street lights. These light sources have much lower luminance values than sunlight, and over long distances they should not affect the VLC network connection [145]. In case the artificial lights are at a relatively short distance from the user connected to the VLC network, they can be blocked by the filter of the optical receiver as long as they have a different direction from the FOV angle.

Due to the attractions and advantages of VLC networks, such as offering greater data security, being less susceptible to interference from external signals, offering greater bandwidth for data transmission, and making efficient use of the frequency spectrum, thereby helping to alleviate congestion in the radio frequency spectrum, multiple studies [140,141,142,143,146,147] have been conducted on the study of VLC networks in outdoor scenarios regardless of the presence or absence of sunlight. The deployment of outdoor VLC networks aims to extend the multiple advantages of this technology to all users, regardless of their locations and scenario. A study of the impact of sunlight noise sources on the packet delivery rate in a VLC network was carried out in [141]. In [142], a time-domain characterization of the VLC channel was performed by conducting experiments in a static outdoor scenario during different times of the day. In [143], a high pass filter was proposed to filter the sunlight intensity in a one-channel VLC experiment. Outdoor VLC network access points could be powered by solar panels or other renewable energy sources, meaning that there would be no additional energy costs during the day. In addition, VLC technology could use directional LEDs with a small light cone that transmit only the desired information and would not be occupied by unnecessary lighting. On the other hand, the VLC network saves energy at night, as the lighting LEDs act as data transmitters, eliminating the need for separate devices for lighting and communication. However, outstanding issues related to interference caused by solar illumination on VLC devices must be resolved. Consequently, several investigations on outdoor VLC networks have focused on this issue [140,141,142,143].

Other external factors that greatly affect the VLC channel include difficult weather conditions such as fog, rain, and snow. Normally, molecules and particles in the atmosphere interact with the light, deflecting the beams and attenuating the transmitted signal. In adverse weather conditions these effects are much more severe, as fog, rain, and snow are made up of larger particles that have a large impact on the range and reliability of VLC. The study conducted in [148] showed that fog has a greater negative impact on VLC; however, the study conducted in [149] showed that dry snow can be even more detrimental to VLC system performance than fog. For this type of scenario in which weather conditions are adverse, it is possible to establish reliable communication up to 15 m using the highest possible modulation scheme [150].

The hybrid VLC/RF networks and their deployment unlock multiple possibilities for IoT applications in various outdoor scenarios [151]. The high-precision communication capabilities of VLC technology make it an effective solution for interfacing with smart sensors that require accurate real-time data transmission. At the same time, the RF network ensures ubiquitous connectivity and uninterrupted communication. Among the applications with potential for innovative systems are smart outdoor streetlights that autonomously adjust to changing traffic patterns, security cameras that detect movements with high precision, and traffic control systems that can effectively manage traffic congestion in real time. With the integration of VLC/RF networks, exciting new possibilities that expand the realms of IoT become a reality [152].

Several authors have started to investigate this topic, and the first steps in the design of hybrid VLC/RF systems in outdoor scenarios can already be found in the scientific literature [153,154,155,156]. In [153], an optimization problem was established to obtain the maximum possible information rate for users through the DC offset and AC peak amplitude of the VLC network access points along with the electrical power supplied to LEDS and relay relays. On the other hand, in [154] the energy efficiency of a Non-Orthogonal-Multiple-Access (NOMA) scheme of hybrid VLC/RF networks with imperfect hybrid channel state information was investigated. It was shown that a non-orthogonal multiple access scheme has higher performance compared to the VLC/RF network in an outdoor scenario where LOS availability is limited. The authors of [155] evaluated a Co-NOMA-based cooperative transmission scheme for hybrid VLC/RF systems in outdoor scenarios where users with weak VLC connections can connect to the network through users with strong VLC connections using the RF network. An increase in the sum rate of the system was demonstrated, as was increased user fairness. Recently, a hybrid FSO-VLC/RF system capable of providing communication services to emergency responders for an outdoor fronthaul network based on a machine learning algorithm was experimentally demonstrated in [156]. A study of outdoor VLC for vehicle-to-vehicle security applications was conducted in [157]. The authors introduce the privacy and security fields in the physical layer of VLC to improve the vehicle-to-vehicle power distribution.

## 4. VLC/RF Hybrid Network Applications

**Figure 10:**
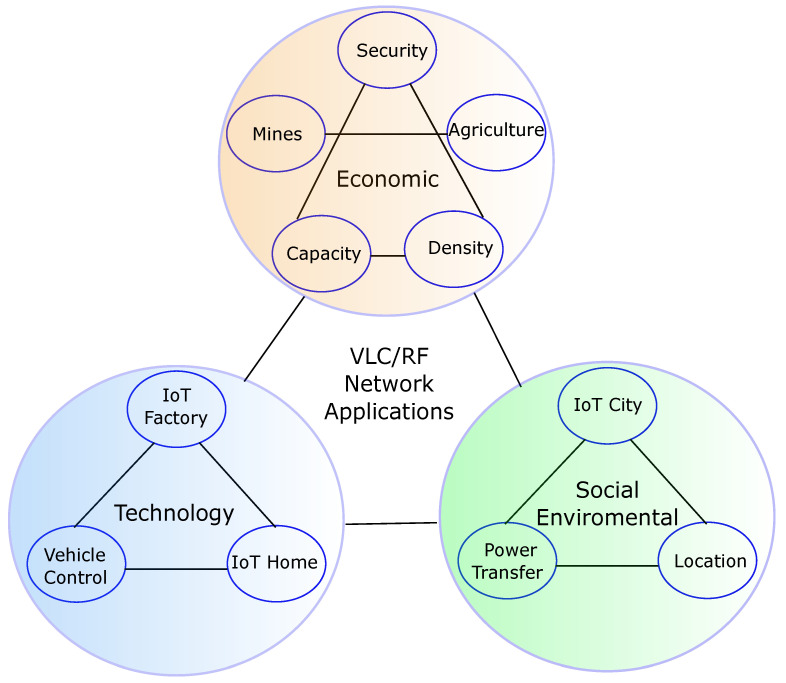
VLC/RF network applications.

Thanks to the varied characteristics of VLC and RF networks and their complementarity, there are a wide range of applications in the technological, economic, and social-environmental fields for these types of networks. VLC/RF hybrid networks have demonstrated great improvements in overall system performance in terms of data rate, load balancing, and lower time delay in both indoor and outdoor scenarios [158]. The implementation cost of these networks is relatively low, and they allow for the reuse of the entire existing lighting infrastructure [159]. The energy efficiency of VLC/RF networks and the possibility of distributing multiple access points without interference or environmental waste can facilitate the decrease of energy consumption and environmental pollution, helping to meet the Sustainable Development Goals for the year 2030 established by the UN [160]. Figure 10 shows an example of the applications of VLC/RF networks and the relationship between them.

### 4.1. Technological Applications

VLC/RF hybrid technology could be a key tool to achieve smart homes. The data speed and security of VLC networks in conjunction with the ubiquity of RF coverage could be used to interconnect all electronic equipment within smart homes. In this way, any command could be executed on any electronic equipment in the house from a mobile terminal or simply by voice, even if far away from the house. The use of a solar panel on the roof of an IoT smart house represents a way to store energy and transmit information at the same time through the VLC network, which could then be transferred to the interior of the house through the RF network. The authors of [161] investigated the applications of hybrid VLC/RF networks in a three-dimensional indoor IoT system, taking into account temperature sensors, indoor air quality sensors, indoor sensors, and humidity sensors.

IoT applications associated with factories and production have attracted the attention of researchers in recent years [103,161,162]. The goal is to use the performance of VLC/RF networks to interconnect production equipment and automation technologies to enable the generation and sharing of information between all equipment. This can enable big data generation to identify certain production patterns and even predict inefficiencies and future events.

Vehicular and traffic control applications on the road are another promising options for the development of automotive technology. Due to the random nature of car traffic on the road, it is necessary to implement a hybrid technology that allows communication between cars and organizes their passage. Several authors have investigated this topic and proposed different vehicle control applications using hybrid VLC/RF networks [163,164,165]. In [163], the authors proposed an outdoor cognitive network with electric vehicles using mixed VLC and RF channels to establish interference-free communication between vehicles. On the other hand, in [164] the authors presented a hybrid VLC/RF cooperative system in vehicle-to-vehicle communication networks and analyzed the performance of the hybrid system in terms of outage probability and bit error rate. The authors of [165] investigated resource management in hybrid VLC/RF systems for wireless communication between vehicles and infrastructures.

### 4.2. Economic Applications

VLC networks offer a great guarantee of security, as the transmitter and receiver must be in the line of sight, the coverage area is very small, and the transmission power is focused only on the light beam. In places where it is necessary to send and receive data that must be protected (e.g., companies, banks, hospitals) the implementation of hybrid VLC/RF networks can provide great security in data transmission [119,127].

In scenarios where there is a high density of users connected to the internet (e.g., stadiums, airports, and parks) the use of multiple RF access points can cause interference and reduce system performance. The use of hybrid VLC/RF networks can solve this problem by reusing the entire existing lighting infrastructure for VLC data transmission. These hybrid networks provide higher data rates, reduce RF interference, and have a very low implementation cost, making them are ideal for high user density scenarios [18,63,89].

Hybrid VLC/RF networks have demonstrated a great improvement in the characteristics and capabilities of wireless communications systems. Research has focused on increasing the data rate [78,84,166] to improve the overall capacity of hybrid system, while other authors have focused on decreasing the system time delay [66,81,167].

Mining communication systems are an application that safeguards the safety of workers in mines and increases the efficiency of their work. In recent years, VLC links have been widely investigated as a complementary technology to RF links for subway mining applications due to their high data rate and freedom from RF interference [168,169]. Subway mines represent a challenging scenario due to their irregular walls and many shadows and dust particles, which produce the scattering phenomenon. For all of these reasons, this industry requires an optimal transverse communication system that can be supplemented by hybrid VLC/RF networks. The use of indoor, motion, and ambient dust particle sensors would be of great help in the implementation of VLC/RF networks in these and similar hostile scenarios.

Technology is a great ally of agriculture, helping to achieve greater efficiency in farming with the objective of supplying the population without depleting the planet’s resources. The implementation of hybrid VLC/RF networks in conjunction with sensors dedicated to agricultural activity can be of great help in monitoring and sending information about events concerning crops and their surroundings [170,171].

### 4.3. Social-Environmental Applications

VLC/RF networks have been presented as a reliable alternative to obtain the location of mobile terminals in deep indoor scenarios (tunnels, mines, and subways) where GPS has no connection. The RF network can be used to obtain the external location of the site, while the VLC network can provide the internal location of the user. Several proposals for positioning applications using VLC/RF networks have been put forward [172,173,174], and a minimum estimation error of 5.8 cm has been demonstrated.

Several authors have investigated the process of power transfer from a VLC network to the nearest users [118,119]. This power transfer allows the energy transmitted by the LED lights to be stored for later use in other transmissions. This application has demonstrated high energy efficiency for hybrid VLC/RF networks, and could have a large impact on overall energy consumption [175,176].

The growth and development of cities is an aspect that has been taken into account in technological research throughout history. The implementation of hybrid networks, especially VLC/RF networks, represents a key parameter to achieve efficient management of each area of the city, thereby improving the life of its inhabitants. Hybrid VLC/RF technology can allow energy optimization, reduce household consumption, organize the movement of vehicles, and increase the flow of mobile traffic, among many other aspects necessary to achieve the development of sustainable cities [83,131,132].

The main advantages of hybrid VLC/RF networks over conventional RF networks are their ability to increase transmission bandwidth and alleviate congestion in RF networks. In addition, these hybrid networks can be deployed and operated satisfactorily in locations where a pure RF network is insufficient. Table 5 comparatively summarizes the performance of VLC/RF and conventional RF networks for the applications proposed in this section. The ⋆ and ★ marks respectively indicate good and excellent performance considering the criteria of previous works and the corresponding performance metrics.

## 5. Challenges and Future Directions for Hybrid VLC/RF Networks

This section discusses important issues facing hybrid VLC/RF networks as well as their future directions, including the use of Software Defined Networking (SDN) platforms, multiple access techniques, the development of new modulation and coding schemes, the design of efficient resource allocation and handover algorithms, the development of security mechanisms, the hybrid channel model, load balancing, uplink communication in VLC, outdoor scenarios, and Optical Camera Communication (OCC).

The use of SDN platforms has been booming in recent years due to their ability to efficiently manage all access points in the network. Several papers have been presented on SDN [177] in which the advantages of its use in resource allocation and network scalability have been demonstrated. The use of SDN platforms in hybrid VLC/Femtocell networks remains in its infancy, and it remains necessary to study this topic in greater depth. The use of SDN platforms in hybrid VLC/RF networks must overcome challenges such as the coexistence of technologies and hybrid topology management [178]. Hybrid VLC/RF networks must be able to interoperate efficiently with existing technologies, which can pose compatibility and interference management challenges. In addition, the management of hybrid topologies may require a more dynamic and flexible approach to ensure optimal connectivity between the two technologies. Therefore, new standards and algorithms for VLC/RF networks on SDN platforms that facilitate interoperability and coexistence of the technologies are required. This can help minimize compatibility issues and manage interference effectively [179].

Supporting user mobility is vital in hybrid networks. The user may move in the scenario with multiple obstacles, which makes the location of the mobile receiver and the coordination mechanism between the LED transmitter and the RF base station a key challenge for multiple access techniques [51]. The CSMA/CA access technique has been presented separately for single network scenarios instead of hybrid networks [180] making a CSMA/CA access technique especially designed for hybrid VLC/RF networks required in order to support user mobility. On the other hand, non-orthogonal multiple access (NOMA) techniques have been presented in recent works as a promising option for multiple access between users via power multiplexing [97,118].

The communication channel is the medium responsible for transmitting information between the transmitter and receiver. Efficient channel modeling is vital because it reduces multi-project and interference effects [40]. It is necessary to optimally design a hybrid channel model that integrates VLC and RF technologies and takes advantage of the benefits provided by each channel independently. The design of hybrid communication channels for VLC/RF technologies is considered a major challenge due to the marked differences in the communication channel of each network. The channel model, modulation and propagation techniques, data rate, transmission capacity, and coverage are among of the characteristics that differentiate VLC and RF networks [45]. Despite this, because VLC and RF networks do not interfere with each other it is possible to design a common channel for both technologies. The correct selection of the modulation techniques of the networks and the synchronization between the channels available for the transmission of both technologies is fundamental to optimizing the design of a hybrid channel [46]. Although several channel designs have been proposed for hybrid VLC/RF networks, it remains necessary to optimally design a hybrid channel model that integrates VLC and RF technologies and takes advantage of the benefits provided by each channel independently.

A technical concern of hybrid VLC/RF networks is to achieve efficient load balancing. The main question is how to allocate users between VLC access networks and RF femtocells. The first step to finding an answer to this question involves solving the problem of joint association and resource allocation [50]. Although several authors have investigated these problems [53,55,56,57,59], several parameters remain to be solved before efficient load balancing can be achieved. It is necessary to delve deeper into issues such as the fair distribution of user load; dynamic allocation algorithms could be developed to ensure this balance [51]. In addition, it is necessary to develop load-balancing algorithms that analyze the state of each base station and dynamically assign users to base stations with less load. This can help to avoid congesting the stations and make the best use of the network resources [53]. During a call session a load balancing mechanism must be performed periodically, and users may need to switch to an AP with better service if they are on the move [70].

Due to the particular characteristics of each technology, it is essential to develop new modulation and coding schemes to improve the performance of hybrid VLC/RF networks [181]. While traditional RF networks have limitations such as electromagnetic interference and signal attenuation, VLC networks are not subject to these limitations and can offer higher transmission rates. However, their sensitivity to physical obstacles and variations in light intensity are relevant challenges. It is necessary to develop modulation and coding schemes to overcome these limitations of hybrid RF/VLC networks. These schemes have to mitigate the adverse effects of interference and attenuation on data transmission in order to improve the quality and reliability of optical communication [182]. These new schemes should be able to adapt to environmental conditions and optimize spectral efficiency to take full advantage of the available bandwidth [183].

Resource allocation and handover are essential to transmit large volumes of data quickly and reliably in hybrid VLC/RF networks [53]. These algorithms are crucial to the effective and balanced allocation of transmission resources such as bandwidth, transmitting power, and processing capacity. Moreover, they ensure continuous and uninterrupted transmission by managing the handover of data between VLC and RF technologies. Resource allocation and handover have a direct impact on the quality of service offered to users by maximizing network capacity and avoiding congestion. In addition, proper resource allocation helps to reduce energy consumption and costs associated with data transmission. Although several algorithms have been proposed for hybrid VLC/RF networks [26,60,66], there is a need to design new algorithms that optimize resource usage and decrease handover latency between networks. The use of tools such as artificial intelligence can be of great help in the evolution towards more efficient algorithms.

The growing dependence on communication technologies and the need to protect the integrity and confidentiality of the information being transmitted makes security mechanisms in hybrid VLC/RF networks of vital importance today [184]. Multiple vulnerabilities and security risks can arise from the use of different communication technologies in hybrid VLC/RF networks. Therefore, it is necessary to develop security mechanisms that protect the privacy of the information transmitted, prevent unauthorized interception of data, and prevent attacks and malicious manipulation. These security mechanisms should include data encryption techniques, user and device authentication, and intrusion detection and prevention. In addition, the integrity of transmitted data must be guaranteed, while access and control policies must be established to ensure that only authorized users can access the network [185,186].

To date, VLC systems lack acceptable uplink performance; for this reason, hybrid networks are forced to use different direct and return communication paths [93,111]. For uplinks, these systems require the beam to be in a fixed direction; thus, shifting the MTs can greatly impair system performance and make the link inadequate. One of the main reasons for this is that smart mobile devices have limited power; it has not yet been possible to integrate a light source for communications [115,116]. Therefore, supporting uplink transmission using VLC is a challenge in the implementation of hybrid systems.

Outdoor scenarios are characterized by their very complex and dynamic communication channel. There are several factors that cause fading of the transmission signal, such as scattering, absorption, non-alignment, turbulence, and sunlight. Sunlight is considered to be the most severe co-channel interference source for VLC networks, and coupled with other sources of signal attenuation results in a very low SNR. Another major challenge for the VLC/RF network is the support of mobility in outdoor scenarios where obstacles and time changes can affect the direct link between the transmitter and receiver. To counteract the adverse effects of sunlight on the VLC channel, the receiver FOV can be optimized by adjusting the receiving angle. However, FOV adjustment is usually a compromise between noise reduction and the possible reception angle [155]. To avoid reducing the receiver FOV too much, in [143] the authors proposed using a high-pass filter to filter the sunlight intensity. In addition, it was proposed to coat the entire area from the optical receiver to the photodiode in order to block unwanted light angles [143]. An optimal design of the communication channel of VLC/RF networks in harsh environments capable of adapting to the existing environmental conditions and enhancing overall system performance is required [153,154].

Hybrid VLC/RF networks could be a key point in long-distance (satellite) communication. This could be achieved through the use of solar panels designed in such a way that they can receive satellite information via sunlight or detecting light from lasers [140]. This is a very interesting open research topic, as it would make sunlight a source of illumination, energy, and data transmission.

Optical camera-to-camera communication technology (OCC) is a branch of visible light communication that has emerged in recent years. OCC communication, like VLC, takes place between an optical source and a camera composed of image sensors. OCC can be implemented in any type of camera, smartphone, or any device that has integrated image sensors [187]. No changes are needed to the current infrastructure of imaging sensors that act as signal receivers; therefore, the complexity and installation cost are very low. Several papers have presented OCC technology as a fundamental key for the development of 5G communication systems in both indoor and outdoor scenarios [188,189,190]. Therefore, it would be very interesting to combine OCC technology with hybrid VLC/RF networks as a way to improve system performance.

Hybrid VLC/RF networks have a great deal of potential for IoT applications. In the future, these networks are expected to be used to improve connectivity in areas with weak RF signals [191]. Additionally, hybrid networks can overcome indoor positioning challenges by combining the high resolution of VLC with the ubiquity of RF networks. Another area in which these networks can be applied is smart agriculture, where VLC can be used to monitor crop growth and RF can be used for data communication. The integration of VLC and RF technologies can lead to the development of more efficient energy-saving solutions. In summary, we anticipate a bright future for hybrid VLC/RF networks in IoT applications in light of their ability to solve many of today’s limitations and offer innovative solutions for a wide range of industries [192].

## 6. Conclusions

Interest in hybrid networks has grown exponentially in recent years due to the limitations of RF systems. In particular, hybrid VLC/RF networks will become relevant for mobile communications beyond 5G and 6G, as such integration meets the required KPIs. Study of the VLC/RF hybrid network framework in an indoor scenario demonstrates the importance of lighting requirements, network load balancing, resource allocation, and network selection for correct deployment of these systems. Optimal design of the hybrid channel model can help to mitigate the effects of interference and multi-path signal loss. Outdoor scenarios are characterized by several factors that cause fading of the transmission signal, such as scattering, absorption, non-alignment, and turbulence. An optimal channel for VLC/RF networks must adapt to harsh environmental conditions and maintain appropriate general performance of the system under such conditions. The study of hybrid VLC/RF networks is very increasing, as demonstrated by the multiple technological, economic, and socio-environmental applications presented for these hybrid systems to date. The combination of VLC and RF has revolutionized the potential of Internet of Things (IoT) systems, unlocking a world of innovative applications, from improving indoor positioning for seamless navigation to enhancing crop growth in precision agriculture. These hybrid networks hold immense potential to create intelligent applications across multiple sectors, paving the way to a more sustainable future. Despite the many advantages of hybrid VLC/RF networks, it is necessary to continue research regarding their incorporation into next-generation mobile networks and their integration with other technologies such as OCC.

## Figures and Tables

**Table 1:** IEEE 802.15.7 (PHY) parameters.

	Data Rate	Light Source	Modulation
PHY I	11.67–266.6 [Kbps]	only one	OOK ^1^, VPPM ^2^
PHY II	1.25–96 [Mbps]	only one	OOK, VPPM
PHY III	12–96 [Mbps]	multiple	CSK ^3^

^1^ OOK: On–off keying. ^2^ VPPM: Variable pulse position modulation. ^3^ CSK: Color Shift Keying.

**Table 2:** Comparison between surveys of hybrid VLC/RF networks; ⋆ means partially analyzed and ★ means deeply analyzed.

Ref.	Year	Lighting Requirements	Load Balancing	Network Selection	Hybrid Channel	Hybrid Topology	Outdoor	Applications
[27]	2016	⋆						
[28]	2018							⋆
[29]	2018	⋆	⋆					
[30]	2019							⋆
[17]	2020		⋆					★
[2]	2021	★	⋆					⋆
[26]	2021			⋆		⋆		⋆
[31]	2022	⋆	⋆					
This paper		★	★	★	★	★	★	★

**Table 3:** Comparison of VLC and RF channel models.

Parameters	VLC Channel Model	RF Channel Model
Model	Lambertian	Hermitian
Propagation mode	Visible light	Electromagnetic wave
Modulation technique	IM/DD, NOMA ^1^	OFDM, OFDM-MIMO, NOMA
Coverage	Minor	Major
Complexity	Simple	complex
Symmetry	No	Yes
Links	Download	Download/Upload
Doppler effect	No	Yes
Interference inter symbols	Minor	Major

^1^ NOMA: Non-Orthogonal-Multiple-Access.

**Table 5:** Summary of VLC/RF network performance for different applications. The ⋆ and ★ marks indicate good and excellent performance, respectively.

Applications	VLC/RF Systems	RF Network
IoT Home [161]	★ (Bandwidth, Latency)	⋆
IoT Factory [162]	★ (Bandwidth, Latency)	★ (Coverage)
Vehicle Control [163]	★ (Latency, Coverage)	⋆
Security [119]	★ (Security, Reliability)	⋆
System Capacity [166]	★ (Energy efficiency)	⋆
Mining Systems [168]	★ (Interference, Connectivity)	★ (Coverage)
agriculture [170]	★ (Interference, Connectivity)	⋆
localization [172]	★ (Underground connectivity)	★ (Coverage, Precision)
Power Transfer [118]	★ (Bandwidth, Latency)	⋆
IoT City [131]	★ (Bandwidth, Latency)	⋆

## Data Availability

Not applicable.

## Abbreviations

The following abbreviations are used in this manuscript:

5G	Fifth Generation
6G	Sixth Generation
AP	Access Point
B5G	Beyond Fifth Generation
CSMA/CA	Carrier Sense Multiple Access with Collision Avoidance
IM/DD	Intensity Modulation/Direct Detection
IoT	Internet of Things
ISO	International Organization for Standardization
ITU-T	Telecommunication Standardization Sector
LED	Light-Emitting Diodes
LiFi	Light Fidelity
LOS	Line-of-Sight
MAC	Medium Access Control
MIMO	Multiple Input–Multiple Output
MMW	Millimeter Waves
NLOS	Non-Line-of-Sight
NOMA	Non-Orthogonal Multiple access
OCC	Optical Camera Communication
OFDM	Orthogonal Frequency Division Multiplexing
PHY	Physical Layer
QoE	Quality of Experience
QoS	Quality of Service
RF	Radio Frequency
SDN	Software Defined Networking
SNR	Signal-to-Noise Ratio
VLC	Visible Light Communication
VLCC	Visible Light Communication Consortium
WiFi	Wireless Fidelity
